# Supplementary material for: SyNDI: synchronous network data integration framework
Source: BMC Bioinformatics. 2018 Nov 6;19:403. doi: 10.1186/s12859-018-2426-5 (PMC6219086; doi:10.1186/s12859-018-2426-5)
Supplement: Supplementary file 5 — Files of the example presented in Additional file 4. This additional file is a zip package that contains all files used in Additional file 4. Detailed descriptions of each of these files are presented in a README file included in this zip package. (GZ 30411 kb) [file 12859_2018_2426_MOESM5_ESM.gz › SyNDITools/Meme2Fimo/meme_4.9.0_4.tar.gz!/meme_4.9.0/website/html/metameme-intro.in]

Meta-MEME v3.3


> Meta-MEME is a software toolkit for building and using
> motif-based hidden Markov models of DNA and proteins. The input to
> Meta-MEME is a set of similar protein sequences, as well as a set
> of motif models discovered by MEME. Meta-MEME combines these
> models into a single, motif-based hidden Markov model and uses this
> model  to search a sequence database for
> homologs.
>
> ---
>
> - Submit data to Meta-MEME for
>   analysis.
> - Learn more about what
>   Meta-MEME does and how it works.
> - Answer your questions with the
>   Meta-MEME FAQ.
> - Read papers about Meta-MEME and
>   related work.
> - Download the Meta-MEME ANSI C
>   source code, as well as program binaries for selected
>   platforms.
> - Browse links to web sites related
>   to motif discovery and hidden Markov modeling.
>
> ---
>
> > Meta-MEME was developed by William Stafford Noble in
> > the Department of Genome
> > Sciences at the 
> > University of Washington and by Timothy Bailey in the
> > Institute for Molecular Bioscience at the
> > University of Queensland, with
> > input from Charles
> > Elkan and Michael
> > Gribskov.
> >
> > Maintenance and development of Meta-MEME is funded by the National Center
> > for Research Resources grant NIH/NCRR R01 RR021692.
> > The Meta-MEME web server is funded by the
> > National Biomedical Computation Resource.
> >
> > Copyright
> > information. Please send comments and questions to Charles
> > Grant at @METAMEME\_CONTACT@.
>
> ---
